# Indications and administration practices amongst medical cannabis healthcare providers: a cross-sectional survey

**DOI:** 10.1186/s12875-019-1059-8

**Published:** 2019-12-14

**Authors:** Jamie Corroon, Michelle Sexton, Ryan Bradley

**Affiliations:** 1 The Center for Medical Cannabis Education, 428 8th Street, Del Mar, CA 92014 USA; 2 Helfgott Research Institute, National University of Natural Medicine, Portland, OR USA; 3 Department of Anesthesiology, University of California San Diego, San Diego, CA USA; 4 Department of Family Medicine and Public Health, University of California, San Diego, La Jolla, CA USA; 5 Australian Research Center on Complementary and Integrative Medicine (ARCCIM), University of Technology Sydney, Ultimo, NSW Australia

**Keywords:** Cannabis, Marijuana, THC, CBD, Cannabinoids

## Abstract

**Background:**

The medical use of cannabis has been legislatively restricted for decades in the US and abroad. In recent years, changing local and national policies have given rise to a community of healthcare providers who may be recommending the medical use of cannabis without the benefit of formal clinical practice guidelines or sufficient training and education. In addition, a citizen science movement has emerged whereby unlicensed and untrained individuals are acting as healthcare provider proxies, offering cannabis-specific clinical care to “patients”. This study sought to characterize the clinical practice characteristics of these provider groups.

**Methods:**

An anonymous, online survey was designed to describe levels of cannabis-specific education, practice characteristics, indications for medical use, dose, administration forms and adverse effects related to cannabis use. The questionnaire was disseminated via professional medical cannabis associations and by word-of-mouth. It was accessed between June 31–December 31, 2018. A self-selecting sample of respondents (*n* = 171) completed the survey.

**Results:**

Formal education or training in the medical use of cannabis was significantly more common among licensed respondents than unlicensed respondents (95.5% vs 76.9% respectively, OR, 6.3, 95% CI, 1.2–32.3, *p* = 0.03). The vast majority (*n* = 74, 83.15%) of licensed respondents reported having recommended cannabis as an *adjunct* to an existing prescription drug. Almost two-thirds (*n* = 64, 71.9%) reported having recommended it as a *substitute*. When delta-9-tetrahydrocannabinol (THC) is the principal therapeutic constituent of interest, vaporization is the most common method of administration recommended (*n* = 94 responses, 71.4% of respondents). In contrast, when cannabidiol (CBD) is the principal therapeutic constituent of interest, oral administration (sublingual or oromucosal absorption) is the most common method (*n* = 70 responses, 71.4% of respondents).

**Conclusions:**

Individuals who recommend the medical use of cannabis appear to be self-generating a community standard of practice in the absence of formal clinical guidelines on dosing, interactions and other characteristics. Reducing barriers to clinical research on cannabis products is needed, not only to better understand their risks and benefits, but also to augment the evidence-base for informing clinical practice.

## Introduction

*Cannabis sativa L. (Cannabis spp. or Cannabis)* is used as both a recreational drug and a botanical medicine. In the United States, the Drug Enforcement Administration (DEA) has considered cannabis (i.e., marijuana) a Schedule I controlled substance since the passage of the Controlled Substances Act in 1970 [1]. Despite this status, and as of this writing, 33 states, the District of Columbia, Guam and Puerto Rico, have legalized the medical use of cannabis [2]. In addition, 11 states, and the District of Columbia, have legalized cannabis for recreational use by adults, with more states likely to follow [3].

This phenomenon is not confined to the US, however. Canada and Uruguay have also legalized adult use of cannabis. In the UK, cannabis products have been moved to Schedule 2, allowing some products to be prescribed as medicines [4]. Cannabis*-*derived medicines have also been authorized by other European countries, including Italy, Croatia, Netherlands, and the Czech Republic [5].

In the US, states with regulated medical cannabis programs allow licensed healthcare providers to qualify patients pursuant to that state’s eligibility criteria. Many, but not all, of these providers also manage these patients’ symptoms and medical conditions over time using cannabis as a medicine. Decades-long restrictions on clinical trials investigating cannabis have thwarted the evidence base upon which clinical practice would typically be informed [6]. As a result, guidance on dosage, methods of administration, contraindications, adverse events, prescription drug interactions, and other important aspects of clinical care has been lacking [7]. While educational resources are available, they may be insufficient for providers to feel confident recommending cannabis as a therapeutic option [8, 9]. For example, 85% of medical students reported not receiving education on the topic at medical school or in residency, and 90% reported not feeling prepared to “prescribe” medical cannabis [9]. In another study, fewer than 20% of Colorado family physicians reported that they had received any information on medical cannabis in their formal education [10]. Only three states have required any training or certification in order to qualify patients for their medical cannabis programs [11].

In the absence of adequately-trained, licensed healthcare providers to advise patients, unlicensed individuals have stepped into the void to serve as healthcare provider proxies, promoting and advocating the use of cannabis as a medicine [12, 13]. In some cases these unlicensed individuals may be employees of cannabis dispensaries. In one study, 94% of such individuals reported providing specific dosing advice to customers, despite their lack of evidence-based training [14].

This scenario has precipitated several potential concerns. Despite good intentions, such individuals may not recognize when considerable medical risk is present [15]. Also, they may not be oriented toward fundamental tenants of medical ethics, including informed consent regarding treatment options [14, 15]. Further, they may be industry-funded, and thus operating with an undisclosed conflict of interest. In some instances, these individuals are effectively practicing medicine without a license and may be making recommendations that conflict with delta 9-tetrahydrocannabinol (THC)-oriented safety guidelines from national organizations, such as the American College of Obstetricians and Gynecologists (ACOG) regarding the use of cannabis in pregnancy [15].

The purpose of this study was to examine and describe a variety of characteristics of both licensed healthcare providers and these unlicensed healthcare proxies who are recommending the medical use of cannabis to their patients or clients. These characteristics span multiple domains and are detailed below.

## Methods

### Survey

The authors developed a novel questionnaire with three distinct aims: 1. To examine and describe the socio-demographic, education, training and clinical practice characteristics of licensed healthcare providers and unlicensed healthcare provider proxies who recommend the medical use of cannabis; 2. To differentiate and elucidate their observations, opinions and practices, and determine whether there is any consensus with regard to indications, dosing, methods of administration, perceived effectiveness, prescription drug substitution and differentiation between approaches utilizing THC and Cannabidiol (CBD) as independent modalities; and 3. To characterize the anecdotal observations, opinions and practices involving the tolerability and safety of cannabis products, including adverse effects, prescription drug interactions, contraindications and cannabis use disorders. The survey was tested for comprehension and clarity of aims using an iterative process by which a select group of individuals completed the questionnaire under supervision by the authors. The final survey consisted of 255 structured questions including yes/no, single response, multiple response and slider/visual analog scale answers.

Respondents were a self-selected convenience sample who accessed the online survey from June 29, 2018 to December 31, 2018 at https://www.medicalcannabis.study. Recruitment strategies included email promotion via several organizations which have emerged to address the need for professional development around the medical use of cannabis, including the American Cannabis Nurses Association (ACNA), the Society of Cannabis Clinicians (SCC), the American Academy of Cannabinoid Medicine (AACM) and HelloMD. Other recruitment strategies included invitations sent via email to licensed cannabis dispensaries, medical cannabis educational and training programs and a list-serve of medical cannabis professionals in Canada. The only participation criterion was answering “Yes” to the following question: “Do you provide services that include recommending and/or advising and/or educating individuals in the use of cannabis for medical or therapeutic purposes?”

Study data were collected and managed using the Research Electronic Data Capture (REDCap) [16] platform hosted at National University of Natural Medicine. REDCap is a secure, web-based application designed to support data capture for research studies, providing: 1) an intuitive interface for data entry (minimum and maximum values were pre-set); 2) audit trails for tracking data manipulation and export procedures; 3) automated export procedures for seamless data downloads to common statistical packages; and 4) procedures for importing data from external sources.

Documentation of informed consent was secured at the start of the survey, initially using an electronic signature and later using a checkbox for affirmation (Y/N) of having read and agreed to the Informed Consent agreement. The only record linking the respondent with their responses was an optional field at the end of the survey where respondents could type their email address in order to be notified when the data were published in a manuscript. Procedures were in accordance with the ethical standards of the Declaration of Helsinki, as revised in 2008. The institutional review board (IRB) of National University of Natural Medicine approved the study protocol.

### Data analyses

To meet Aims 1–3, descriptive statistics were calculated, including frequencies, simple proportions, means and standard deviations, to describe socio-demographics, clinical practice characteristics, education and training, perceived effectiveness, adverse effects, contraindications, dosage and other attributes. To detect differences between provider types, bivariate comparisons were conducted using frequency procedures and chi-square or binary logistic regression where appropriate. Data analyses were conducted using SAS University Edition (SAS 9.4) (SAS Institute Inc., Cary, NC). An alpha threshold of alpha = 0.05 was applied for all unique significance tests. Bonferroni corrections were applied to pairwise comparisons where relevant. Figures were produced using GraphPad Prism, Version 6.

## Results

### Socio-demographic characteristics

A total of 171 respondents completed the survey, including fourteen different types of licensed providers (including “Other”) and four different types of unlicensed healthcare provider proxies (including “Other”) (See Table 1). The overall survey was comprised of respondents from 22 U.S. states and 12 foreign countries. Unlicensed proxies comprised less than 15% of the sample (*n* = 25, 14.8% of respondents). The most frequently reported type of unlicensed individual was “Non-employee - Cannabis Consultant/Specialist/Educator”. Only three respondents identified as “Employee - Cannabis retailer”.

**Table 1:** Socio-demographic and other characteristics of survey respondents, 2018 (*n* = 171)

	*n* (%)
Gender	
Male	57 (34.5)
Female	108 (65.5)
Decline to State	0 (0)
Missing	6
Age (Mean, SD)	53.2 (11.9)
Race/Ethnicity	
Caucasian	139 (84.2)
Other	26 (15.8)
Missing	6
Geography	
United States	140 (87.0)
Other	21 (13.0)
Missing	10
Geography – U.S. States (Top 5)	
California	37 (29.1)
Massachusetts	10 (7.9)
Illinois	10 (7.9)
Oregon	8 (6.3)
Colorado	7 (5.5)
Other	47 (37.1)
Missing	44
Provider Type (Licensed/Unlicensed)	
Licensed	144 (85.2)
Unlicensed	25 (14.8)
Missing	2
Provider Type (Licensed)*	
Registered Nurse (RN)	66 (46.5)
Medical & Osteopathic Doctor (MD, DO)	39 (27.5)
Other	27 (19.0)
Naturopathic Doctor (ND)	12 (8.5)
Physician’s Assistant & Nurse Practitioner	10 (7.0)
Missing	2
State Regulatory Status	
State regulated MMJ** program	133 (87.5)
No state regulated MMJ program	19 (12.5)
Missing	19
MMJ Authorizations	
Provides	58 (38.7)
Does not provide	92 (61.3)
Missing	21

Key: * Does not sum to 144 or 100% because providers can hold more than one license type. ** MMJ = Medical Marijuana

### Education & Training

The majority of respondents (*n* = 94, 93.1%) reported having received at least some formal education or training in advising or educating patients or clients in how to use cannabis for medical purposes. Receipt of formal education or training was more common among licensed than unlicensed respondents (95.5% vs 76.9% respectively, OR, 6.3, 95% CI, 1.2–32.3, *p* = 0.03), This finding was not statistically significant after applying Bonferroni correction, however.

Less than one third (*n* = 25, 28.4%) of licensed respondents reported receiving specific education about the endocannabinoid system in their formal medical training, or specific education in recommending cannabis in clinical situations. Respondents reported participating in both CME-certified and non-CME-certified education (61.7% for both). Despite the fact that many respondents (*n* = 57, 56.4%) reported that the available education was insufficient, roughly three-quarters (*n* = 71, 75.5%) felt it adequately prepared them to advise patients and clients in the medical use of cannabis, and that the education was free of conflicts of interest (*n* = 68, 72.3%). There were no statistically significant differences between licensed and unlicensed respondents in relation to conflicts of interest in available education and training.

### Clinical practice characteristics

Almost 60% of respondents (*n* = 90, 59.2%) reported recommending and/or advising and/or educating individuals in the use of cannabis for medical or therapeutic purposes for more than 2 years. The number of “visits” per patient reported by licensed respondents was fairly evenly distributed across the responses with approximately one quarter reporting 1–2 visits per patient (*n* = 31, 24.2%) and another quarter reporting more than 5 visits per patient (*n* = 33, 25.8%). Roughly a third (*n* = 43, 33.1%) of licensed respondents offer their services using telemedicine.

Just under half (*n* = 59, 45.7%) of licensed respondents reported recommending cannabis to a majority of their patients and clients, while 13.2% (*n* = 17) reported recommending it in each and every visit. Almost three-quarters (*n* = 110, 73.3%) of all respondents reported that the majority of their patients or clients were inexperienced and had never used cannabis before.

Almost 90% of all licensed respondents (*n* = 114, 87.7%) reported practicing in a state, or country, that affords their patients legal access to cannabis products. A minority (*n* = 58, 38.7%) reported providing written authorizations qualifying patients for a state or country regulated medical cannabis program (See Table 1).

### Methods of administration

Patient and client preference for methods of administration in decreasing order were: oral (ingestion: *n* = 78, 69.6% and sublingual or intra-oral: *n* = 71, 63.4%); topical or transdermal (*n* = 65, 58.0%); and inhalation (vaporizing: *n* = 59, 52.7% and smoking: *n* = 44, 39.3%). It is common for patients and clients to utilize more than one method of administration.

When THC is the principal therapeutic constituent of interest, vaporizing was the most frequent method of administration reported (*n* = 94 responses, 71.4% of respondents). In contrast, when CBD is the principal therapeutic constituent of interest, oral methods of administration were more frequently reported (sublingual or oromucosal absorption: *n* = 70 responses, 71.4% of respondents; ingestion: *n* = 66 responses, 67.3% of respondents).

### Clinical effectiveness

**Fig. 1:**
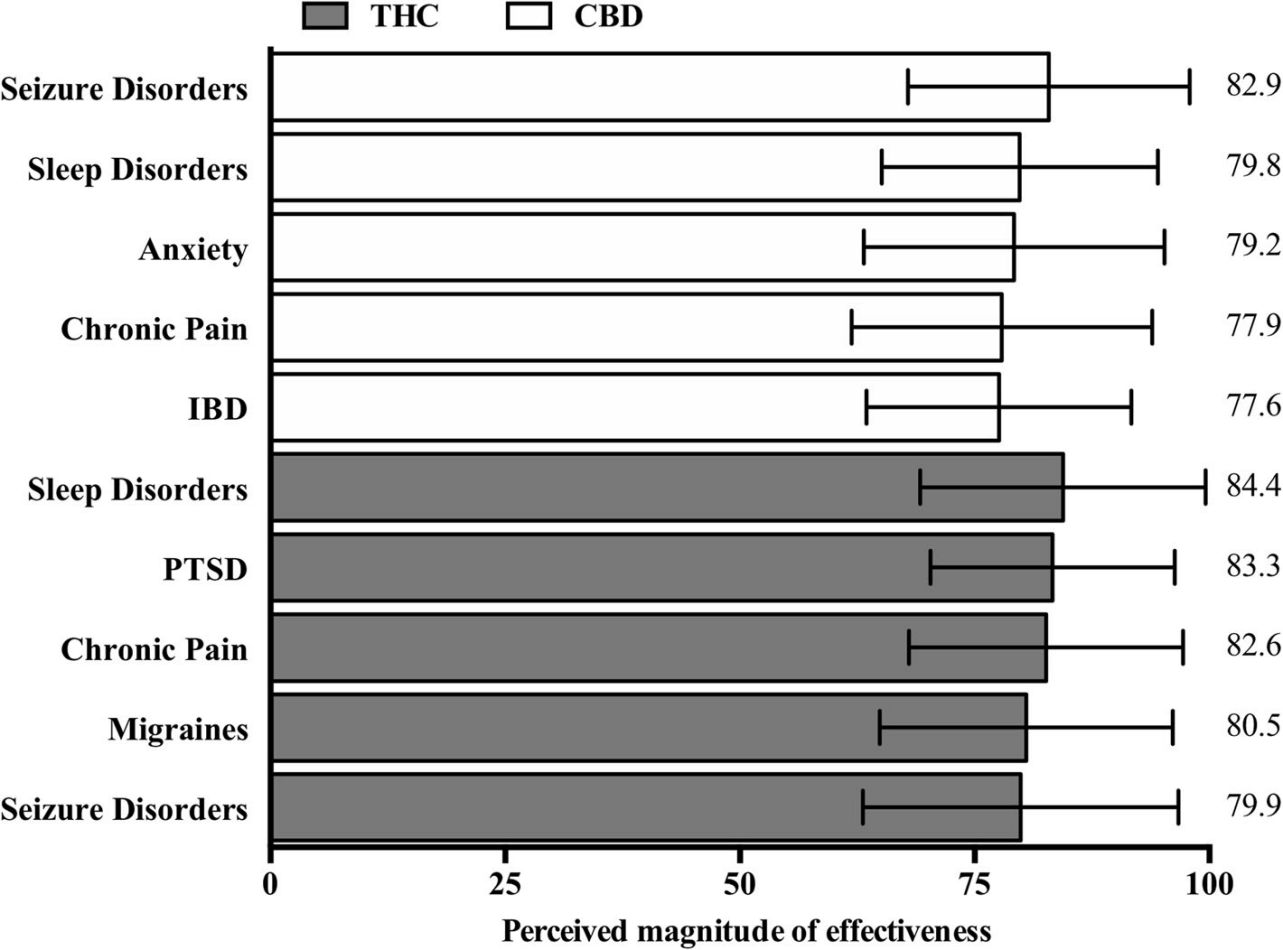
Top 5 most frequently selected symptoms/conditions for which respondents perceive effectiveness by phytocannabinoid, ranked by perceived effectiveness score (in descending order). Respondents were asked, “In your experience, which symptom(s)/condition(s) are most effectively treated with either CBD or THC-dominant cannabis flower and products? (Check all that apply)”. Bars reflect the mean respondent-perceived effectiveness score treated with either CBD (white) or THC (grey). Respondents rated each symptom/condition using the following scale: 0–32 = minimally effective, 33–65 = moderately effective, > 66 = extremely effective. Error bar is the standard deviation of effectiveness score; mean value reported on the right side of the graph. IBD: inflammatory bowel disease; PTSD: posttraumatic stress disorder

Mean provider perceived effectiveness scores for symptoms/conditions treated with cannabis are presented in Fig. 1 below for both CBD-dominant and THC-dominant flower and products.

**Fig. 2:**
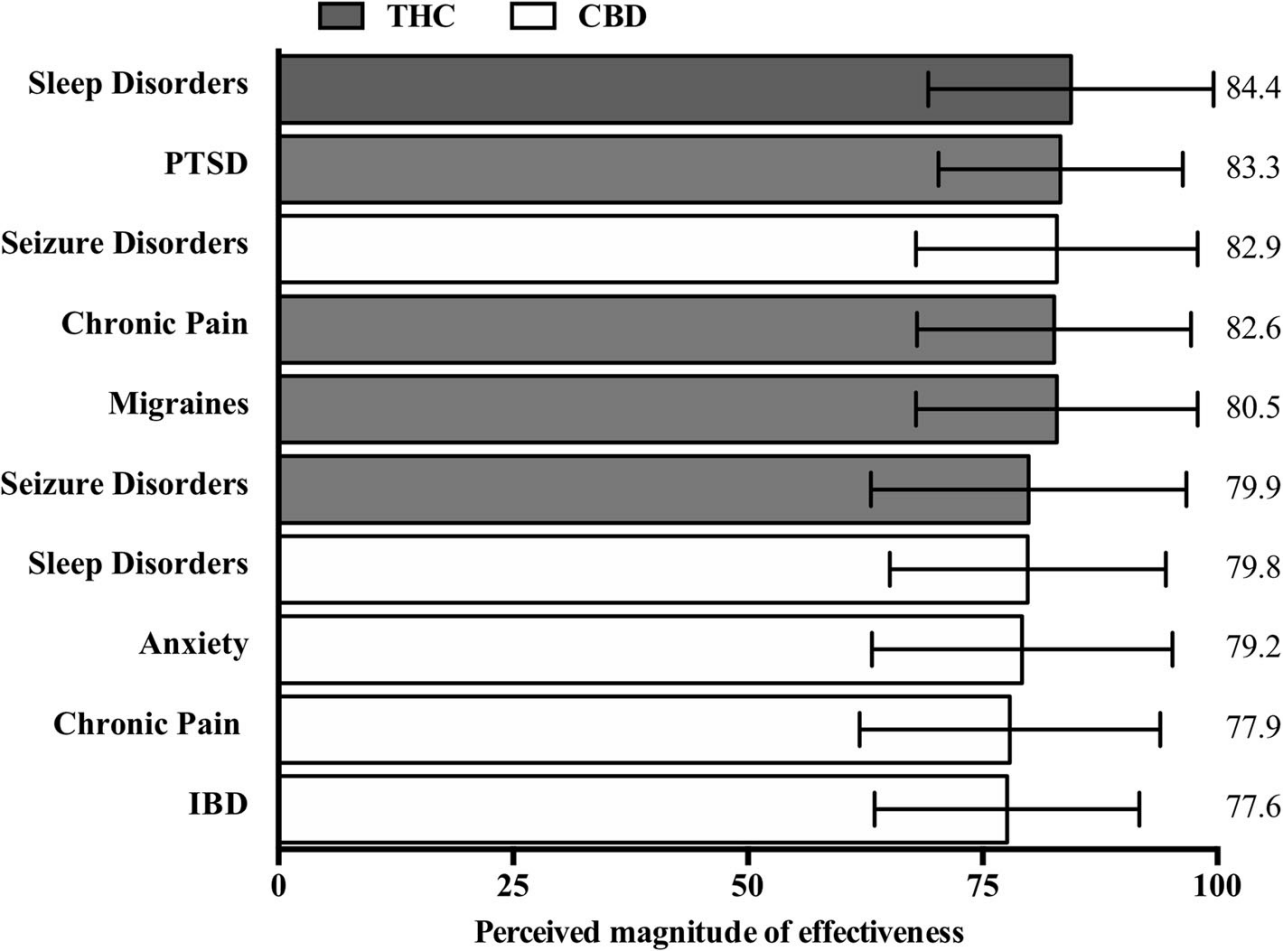
Perceived effectiveness of phytocannnabinoids by symptom/condition, ranked by perceived effectiveness score (in descending order). Respondents were asked, “In your experience, how effective are either CBD or THC-dominant cannabis flower and products at treating [symptom/condition]?”. Bars reflect the mean respondent-perceived effectiveness score treated with either CBD (white) or THC (grey). Respondents rated effectiveness for each symptom/condition using the following scale: 0–32 = minimally effective, 33–65 = moderately effective, > 66 = extremely effective. Error bar is the standard deviation of effectiveness score; mean value reported on the right side of the graph. PTSD: posttraumatic stress disorder; IBD: inflammatory bowel disease

The symptoms/conditions with the 10 highest Mean Effectiveness Scores across both THC-dominant and CBD-dominant flower and products are presented in Fig. 2 below in descending order.

### Dosing

For inhaled CBD-dominant flower and products, respondents selected “I advise titrating up from a low dose” as the most frequent dosing response for 18 out of the 25 different symptoms/ conditions (exceptions: anorexia, asthma, CVD, chronic pain, hypertension, infertility, stroke). For orally administered CBD, respondents selected this response for 21 out of the 25 (exceptions: CVD, infertility, anorexia, infertility). For both inhaled and orally administered THC-dominant flower and products, respondents selected “I advise titrating up from a low dose” as the most frequent dosing response for 23 out of the 25 different symptoms/conditions (inhalation exceptions: anorexia, infertility; orally administered exceptions: CVD, infertility).

**Fig. 3:**
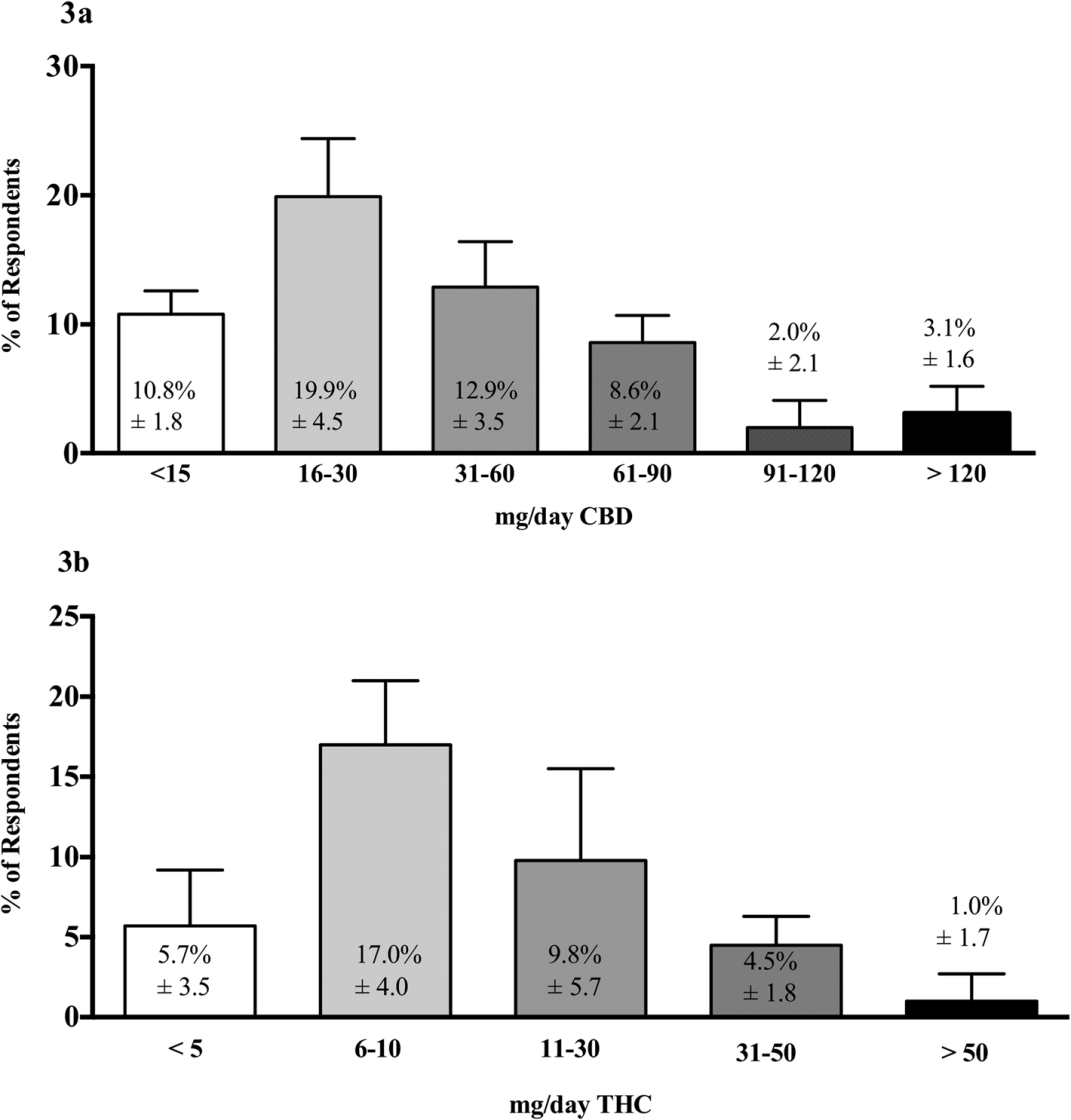
Average daily dose of phytocannabinoids for the top 5 symptoms/conditions (Ingestion). Respondents were asked, “When recommending either CBD or THC as the principal therapeutic constituent, what daily dose of CBD/THC do you typically recommend for the following symptom(s)/condition(s) when ingestion is the method of administration?”. Respondents only answered for conditions that they treat and were not required to record a response for every symptom/condition. Bars reflect the average mg/day dose of either: a) CBD, most commonly used by respondents for anxiety, arthritis, fibromyalgia, sleep disorders and HA/migraine (Top 5 by frequency); or, b) THC, most commonly used for chronic pain, fibromyalgia, arthritis, sleep disorders and anorexia (Top 5 by frequency). The percent response for each dose is numbered inside the bar graph with the standard deviation (CBD, n = 99; THC, n = 132). (% does not =100% as respondents could choose other options such as “I don’t give quantitative dosing advice”)

Quantifiable dosing information for the symptoms/conditions for which THC and CBD-dominant flower and products were most frequently reported being effective are highlighted in Fig. 3a for CBD and 3b for THC. Dosing information is only provided for oral administration.

The most frequently selected quantifiable dosing response for oral administration for treating cancer was > 120 mg per day (15.1% of respondents) for CBD and > 50 mg per day (17.5% of respondents) for THC.

### Hemp versus marijuana

More than two-thirds (*n* = 68, 68.7%) reported recommending marijuana-derived CBD products over hemp-derived CBD products. Almost half (*n* = 49 respondents, 49.49%) reported that “based on observation and experience” marijuana-derived CBD products were more effective. Approximately 30% (*n* = 29 respondents, 29.3%) reported not knowing which was more effective. When asked to select the most common intended effects when recommending CBD, respondents predominantly reported intending to achieve reductions in pain and inflammation (91.9 and 89.9% of respondents respectively), as well as anxiety (84.8% of respondents).

### Adverse effects

Respondents were offered 16 unique adverse effect responses for THC and CBD, plus “Other” (See Appendix A, 7–8. for list of adverse effects). When asked if patients or clients reported adverse effects (Y/N) when using THC and/or CBD-dominant flower and products, roughly twice as many respondents answered affirmatively when referring to THC (Yes: 65.5% vs 33.3% respectively). Respondents also reported a greater frequency of adverse effects with treatments involving THC as compared to CBD (*n* = 386 total adverse effects reported by 72 respondents for THC; *n* = 74 reported by 31 respondents for CBD).

The most frequently reported adverse effect of CBD-dominant flower and products (i.e., Fatigue/Sedation, *n* = 21 respondents, 67.7% of respondents) was equal in frequency to the 9th most frequently reported adverse effect of THC (i.e., Tachycardia, heart palpitations, *n* = 21 responses, 29.2% of respondents) (See Table 2).

**Table 2:** Top 10 adverse effects – THC (*n* = 72 respondents) & CBD (*n* = 31 respondents)

		THC		CBD
Rank	Adverse effect	% of Respondents	Adverse effect	% of Respondents
1	Fatigue, sedation	65.3	Fatigue, sedation	67.7
2	Anxiety	63.9	Other	45.2
3	Dry mouth and eyes	54.2	Headache	22.6
4	Dizziness	52.8	Anxiety	16.1
5	Appetite stimulation	37.5	Dizziness	12.9
6	Impaired concentration	36.1	Dry mouth and eyes	12.9
7	Dysphoria	34.7	Impaired concentration	12.9
8	Impaired memory	31.9	Nausea, vomiting	12.9
9	Tachycardia, heart palpitations	29.2	Tachycardia, heart palpitations	9.7
10	Altered sense of time	26.4	Appetite stimulation	6.5

*This is a multiple response variable (i.e., Check all that apply). Percent of respondents, not responses. Does not sum to 100%

Approximately one-third (n = 31, 33.3%) of respondents reported that their patients or clients experienced adverse effects from isolated CBD or CBD-dominant flower or products. Interestingly, the difference in frequency between the most frequently reported adverse effect of CBD (i.e., Fatigue/Sedation) and the second most frequently reported adverse effect of was substantial (i.e., Headache) (*n* = 21, 67.7% vs *n* = 7, 22.6%, respectively). The most frequently reported “Other” adverse effect was diarrhea (*n* = 3).

### Contraindications

Respondents were asked about absolute contraindications for recommending THC and CBD-dominant flower and products. Respondents were offered 10 unique absolute contraindication responses, plus “I don’t know”, “No” and “Other” (See Appendix A, 9 for list of contraindications.).

**Fig. 4:**
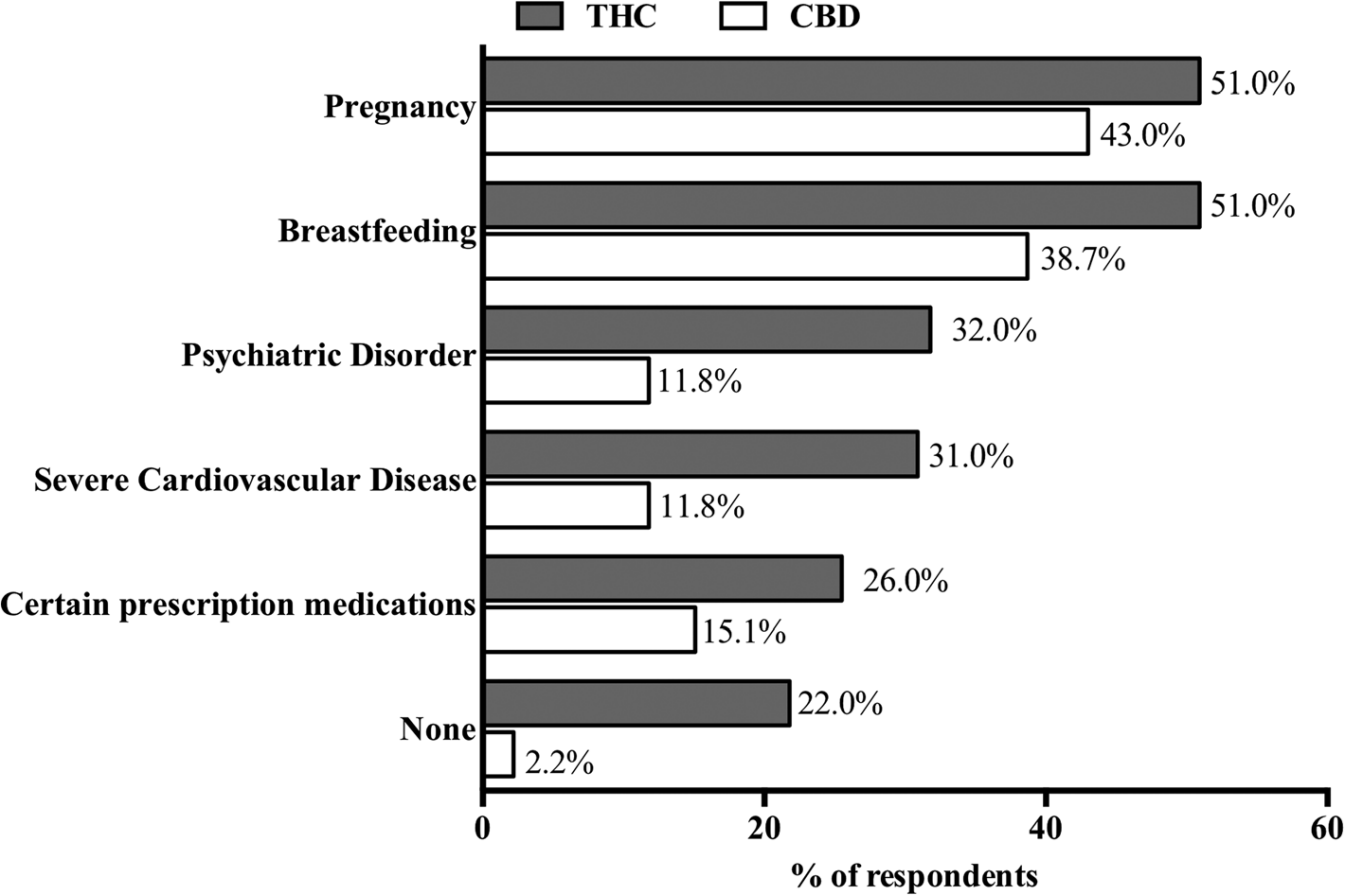
Top 5 absolute contraindications for recommending THC or CBD. Respondents were asked, “Are any of the following absolute contraindications for recommending CBD/THC-only or CBD/THC-dominant cannabis products (Check all that apply)?” The bars indicate the % of respondents who selected each contraindication. The percentage is labeled at the end of each bar (THC, n = 110; CBD, n = 93)

Three hundred thirty-four responses for absolute contraindications were recorded for THC-dominant flower and products by 110 different respondents, while 191 were recorded for CBD-dominant flower and products by 93 different respondents (See Fig. 4 below). This represents 3.0 absolute contraindications per respondent for THC-dominant flower and products as compared to 2.1 for CBD-dominant flower and products. The most commonly reported “Other” contraindication for CBD was concurrent use with anti-coagulant therapy.

Over half of respondents (*n* = 49, 55.7%) reported that “there are clinically significant interactions (pharmacokinetic/dynamic) between cannabis and certain prescription drugs”.

### Adjunctive therapy & prescription drug substitution

**Fig. 5:**
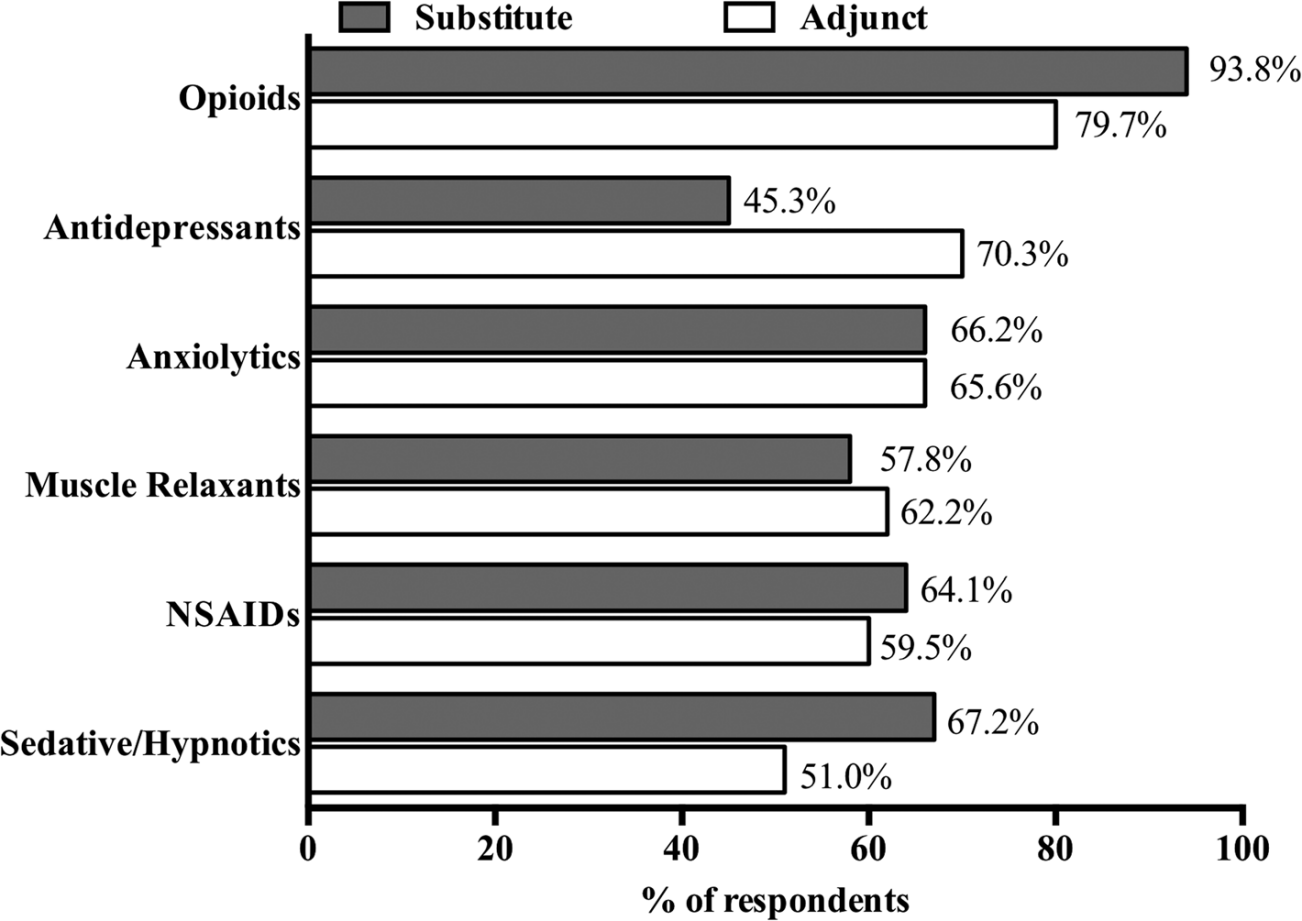
Percent reporting that patients/clients use cannabis as an adjunctive or drug substitution therapy. Respondents were asked, “If you have ever recommended cannabis as a complementary therapy to [substitute for] a prescription drug, what class/category of drug(s) did you intend to complement [substitute] with cannabis (Check all that apply)?” Bars reflect the % of respondents who selected each class/category. The percentage is labeled at the end of each bar. The gray bar indicates respondents reporting using cannabis as a substitute (n = 64). The black bar indicates respondents reporting using cannabis as an adjunct (n = 74)

The vast majority (*n* = 74, 83.15%) of licensed respondents reported having recommended cannabis as an adjunctive therapy to an existing prescription drug. Figure 5 below summarizes the most frequently reported classes/categories of drugs to which cannabis was added. The three most frequently reported outcomes when adding cannabis as an adjunctive therapy were: “Improvement of symptoms” (*n* = 63 responses, 85.1%); “Decrease in dose of prescription medication” (*n* = 55, 74.3%); and “Discontinuation of prescription medication” (*n* = 37, 50.0%).

Almost three-fourths (*n* = 64, 71.9%) of licensed respondents reported having recommended cannabis as a substitute for an existing prescription drug. Figure 5 also summarizes the most frequent classes/categories of drugs for which cannabis was recommended as a substitute. When asked if the substitution was sustained, 64.1% of licensed respondents reported “Yes” (*n* = 41, 64.1%). Almost 30% reported “Not sure/Lost to follow-up” (*n* = 19 respondents, 29.7%) and 6.3% reported “No” (*n* = 4, 6.25%).

### Cannabis use disorders

Licensed respondents were asked, “Have any of your patients and clients ever developed a use disorder involving cannabis as defined by the Diagnostic and Statistical Manual of Mental Disorders, 5th Edition?”. Almost 90% (*n* = 78, 88.6%) reported observing cannabis withdrawal syndrome, while less than 6% (*n* = 5, 5.7%) reported observing cannabis use disorder.

## Discussion

To our knowledge, this is the first survey to report community, practice-based observations, opinions and practices of healthcare providers and unlicensed healthcare proxies who are actively advising patients or clients in the medical use of cannabis. These results suggest that there is consensus among these individuals regarding the need for more and higher quality education, and a lack of consensus regarding dosing of phtyocannabinoids and contraindications for medical use. These data may not be generalizable to practitioners across the globe, however. The sample was largely comprised of Caucasian females in their mid-50s years of age who provide these services in states with regulated medical cannabis programs. Licensed Registered Nurses were the single largest provider type, although more than 25% were Medical Doctors. Unlicensed healthcare proxies comprised less than 15% of the sample.

Despite generally high rates of participation in formal education and training, the present observations indicate that available education may be insufficient, consistent with previous reports [8, 9]. This insufficiency, combined with the lack of prospective clinical research, may be creating a wide disparity in observations, opinions and practices among providers, who have been passively forced to create a community standard of practice in the presence of limited evidence and in absentia of substantive formal practice guidelines.

Respondents indicate that approximately 73% of their patients and clients report being naïve to cannabis. These individuals are presumably in need of medical advice, which would be optimally delivered by individuals possessing sufficient training, education, and legal authority. Forty-five percent report recommending cannabis to a majority of their patients or clients, but only a small portion (13%) recommend cannabis at every visit. These data support our observations that when States legislate the medical use of cannabis, providers may seek to capitalize by forming a medical cannabis specialty practice. This is very different from Great Britain, for example, where doctors are only authorized to prescribe cannabis products within their own specialty, and only when other established options for treatment have been exhausted [15, 17]. Individuals naïve to cannabis may be seeking expert advice more often than non-naïve users. Further, the ongoing stigma from the medical community at large may drive such individuals to unlicensed healthcare provider proxies for advice.

In this study, reports of preferences for oral methods of administration for CBD-dominant products, and inhalation methods for THC-dominant products, approximate reports of cannabis users themselves [18, 19]. Administration methods are particularly important with cannabis because phytocannabinoid pharmacokinetics are greatly influenced by route of administration. For example, compared to inhalation, enteral methods are marked by low bioavailability of phytocannabinoids, due to poor aqueous solubility and extensive metabolism [20, 21]. Also, adverse effects occurring as a direct result of the method of administration (e.g., respiratory irritation via inhalation) need to be taken into account.

The perceived effectiveness of CBD for chronic pain, anxiety and sleep disorders, and THC for pain, sleep disorders and anorexia, is corroborated by cross-sectional studies of cannabis users [18, 19, 22]. However, this study is the first to describe common dosing parameters currently being utilized in cannabis-centric clinical practice, both for oral and inhaled methods of administration. For many symptoms/conditions, respondents most often recommended between 16 and 45 mg of CBD per day orally, and between 6 and 10 mg of THC per day orally, except for chronic pain where the reported dose of THC was typically higher (i.e., 11–30 mg). Recommending higher milligram doses of THC for chronic pain, as compared to most other symptoms/conditions, is consistent with at least one randomized controlled trial of an oral cannabis preparation (i.e., Nabiximols) where participants with severe, cancer-related pain titrated up to approximately 30 mg of THC and CBD each daily [23].

Chronic pain was the most frequently selected indication for using CBD. This observation is consistent with a cross-sectional study of cannabis users in one study [22], but interesting given the limited pre-clinical and clinical evidence supporting the analgesic effects of CBD [24–27]. More research into the analgesic effects of CBD is needed, including efficacious dosing and a determination of the types of pain for which CBD may be effective.

The present observations illustrate how clear and effective dosing information for individual phytocannabinoids is both relatively scarce and yet very important. These data suggest that there is no clear consensus on specific dosages for specific conditions, but that the range of doses utilized in clinical practice is not excessive. Also, it is not unrealistic to speculate that dosing may be driven by the products available in the geographic area of practice.

Methods of estimating the dose of phytocannabinoids from inhaled cannabis are extremely limited. While the bioavailability is much greater than orally administered products [28], milligram amounts of THC and/or CBD are typically not available on labels of inhaled products. In addition, meaningful amounts of these compounds may be lost during combustion (i.e., smoking), or in “side stream smoke” or vapor [29].

The greater frequency of adverse effects associated with THC, as compared to CBD, is expected given its intoxicating potential, and the rates of adverse effects observed in clinical trials [30]. Despite favorable reports of the safety and tolerability of CBD [31], it is not without adverse effects [32]. A high percentage of respondents (67.7%) reported “Fatigue/Sedation” as an adverse effect. Not surprisingly, “Fatigue/Sedation” is often the most commonly reported adverse effect in clinical trials investigating isolated CBD preparations [33–35]. These analyses did not attempt to associate adverse effects with dose, despite the fact that adverse effects are known to be dose dependent [7, 23].

To our knowledge, this is the first survey to report community opinions that marijuana-derived CBD products may be more clinically effective than hemp-derived CBD products. This contention is commonplace among industry stakeholders invested in marijuana-derived CBD products, yet unsubstantiated by existing scientific studies. It should be noted that the methodological constraints of the present study limit its ability to effectively substantiate this claim. Differences in effects between marijuana-derived and hemp-derived products may be due to levels of THC, which can be significantly lower in hemp-derived products [36].

Use of THC and CBD while pregnant or breastfeeding was the most commonly reported absolute contraindication. This community practice is congruent with guidelines from the ACOG, the American Academy of Pediatrics (AAP) and the Centers for Disease Control and Prevention (CDC) [37–39]. Interestingly, almost half of respondents do not consider THC to be contraindicated during pregnancy and while breast feeding, and therefore may either not be aware of the ACOG/AAP/CDC guidelines or are choosing not to follow them.

This study supports observations from previous studies, showing that healthcare providers are recommending the use cannabis as both an adjunctive and substitute therapy for a variety of prescription medications, most commonly opioid and non-opioid analgesics, anti-depressants and anxiolytics [18, 40, 41]. The perspective of those surveyed here is that the addition and/or substitution of cannabis is allowing individuals to reduce symptoms, decrease the dose, and/or discontinue use of prescription medications. Some medications to which cannabis is being added to, or substituted for, have a narrow therapeutic window or are prone to interactions. This area should be an urgent research priority.

### Strengths & Limitations

This study has several strengths, including: a multi-state recruitment approach; dissemination via representative organizations, as well as, through grassroots word-of-mouth increasing generalizability; inclusion of, and discrimination between, both licensed and unlicensed respondents; differentiation between methods of administration, dose and form (flower or processed product) of cannabis-derived products.

Nevertheless, the study has several limitations. The population was a self-selected convenience sample, and as such, may not be representative of the general population. At the time of writing the questionnaire, we estimated 1000 healthcare providers. A sample size of 95 would provide 95% confidence in detecting a 20-point difference between proportions powered at 80%. The sample size estimate was based on an approximation of the membership of the professional organizations that were engaged to assist with recruitment, as well as an estimate of the size of the authors collective professional networks thru which the questionnaire was distributed. An estimate of licensed dispensaries in San Diego, CA and Portland, Oregon was also incorporated. Also, providers with favorable opinions of, or experiences with, cannabis may be more likely to have responded. When uncoupled, observations herein are more likely attributable to licensed respondents, as opposed to unlicensed respondents, due to the predominance of respondents in that group. Since the survey was primarily circulated via the internet, providers with limited technology access may be underrepresented. Beyond the solicitation of provider opinions on clinical efficacy, the survey did not attempt to discriminate between hemp-derived and marijuana-derived products, which may have differing chemical constituents, particularly with regard to THC, and therefore different therapeutic and/or adverse effects. Nor did they survey attempt to inquire observations, opinions or practices related to terpenes/terpenoids, which are also known to have biological activity [42]. No mechanism for identifying repeat respondents was incorporated into the survey. Although results were examined manually, it is possible (albeit unlikely because there was no incentive to do so) that repeat respondents may have distorted the results. Finally, due to a technical glitch, data on dosing was not captured for oral dosing of CBD for chronic pain and inhalation dosing of THC for sleep disorders.

## Conclusion

Collectively, the results reported here indicate that licensed healthcare providers and unlicensed healthcare provider proxies are recommending the medical use of cannabis to their patients or clients, independent of state regulations and licensure, and despite insufficient education and training. These individuals are seeking out training that was not provided in their formal education and generally report safe and effective health outcomes. Among these outcomes include symptom reduction and a decreased reliance on prescription medications. A self-generating community standard of practice may be emerging in the absence of formal clinical guidelines. Reducing barriers to formal clinical research on cannabis products is needed, not only to better understand their risks and benefits, but also to augment the evidence-base for informing clinical practice.

## Data Availability

The questionnaire, datasets used and/or analyzed during the current study are available from the corresponding author on reasonable request.

## Abbreviations

AACM: American Academy of Cannabinoid Medicine
AAP: American Academy of Pediatrics
ACNA: American Cannabis Nurses Association
ACOG: American College of Obstetricians and Gynecologists
CBD: Cannabidiol
CDC: Centers for Disease Control and Prevention
CVD: Cardiovascular Disease
DEA: Drug Enforcement Administration
IRB: Institutional Review Board
REDCap: Research Electronic Data Capture
SCC: Society of Cannabis Clinicians
THC: delta 9-tetrahydrocannabinol
